# RETRACTION: Effect of Topical Antibiotics on the Prevention and Management of Wound Infections: A Meta‐Analysis

**DOI:** 10.1111/iwj.70186

**Published:** 2025-01-12

## Abstract

**RETRACTION**: ZhangM. , FengH. , GaoY. , GaoX. , and JiZ. , “Effect of Topical Antibiotics on the Prevention and Management of Wound Infections: A Meta‐Analysis,” International Wound Journal 20, no. 10 (2023): 4015–4022, 10.1111/iwj.14290.37429583 PMC10681525

The above article, published online on 10 July 2023, in Wiley Online Library (http://onlinelibrary.wiley.com/), has been retracted by agreement between the journal Editor in Chief, Professor Keith Harding; and John Wiley & Sons Ltd. Following an investigation by the publisher, all parties have concluded that this article was accepted solely on the basis of a compromised peer review process. The editors have therefore decided to retract the article. The authors disagree with the retraction.

**RETRACTION**: M. Zhang , H. Feng , Y. Gao , X. Gao , and Z. Ji , “Effect of Topical Antibiotics on the Prevention and Management of Wound Infections: A Meta‐Analysis,” International Wound Journal 20, no. 10 (2023): 4015–4022, 10.1111/iwj.14290.37429583 PMC10681525

The above article, published online on 10 July 2023, in Wiley Online Library (http://onlinelibrary.wiley.com/), has been retracted by agreement between the journal Editor in Chief, Professor Keith Harding; and John Wiley & Sons Ltd. Following an investigation by the publisher, all parties have concluded that this article was accepted solely on the basis of a compromised peer review process. The editors have therefore decided to retract the article. The authors disagree with the retraction.

